# Auditory deficits in infants at risk for dyslexia during a linguistic sensitive period predict future language

**DOI:** 10.1016/j.nicl.2021.102578

**Published:** 2021-02-01

**Authors:** Maria Mittag, Eric Larson, Maggie Clarke, Samu Taulu, Patricia K. Kuhl

**Affiliations:** a Institute for Learning & Brain Sciences, 1715 Columbia Road N, Portage Bay Building, Box 357988, University of Washington, Seattle, WA 98195-7988, USA; b Department of Physics, 1715 Columbia Road N, Portage Bay Building, Box 357988, University of Washington, Seattle, WA 98195-7988, USA

**Keywords:** Dyslexia, Marker, Infant, Auditory, MEG

## Abstract

Developmental dyslexia, a specific difficulty in learning to read and spell, has a strong hereditary component, which makes it possible to examine infants for early predictors of the condition even prior to the emergence of detectable symptoms. Using magnetoencephalography (MEG), we found smaller and shorter neural responses to simple sounds in infants at risk for dyslexia at 6 as compared to 12 months of age, a pattern that was reversed in age-matched controls. The findings indicate atypical auditory processing in at-risk infants across the sensitive period for native-language phoneme learning. This pattern was robust and localized to the same cortical areas regardless of the modeling parameters/algorithms used to estimate the current distribution underlying the measured activity. Its localization to left temporal and left frontal brain regions indicates a potential impact of atypical auditory processing on early language learning and later language skills because language functions are typically lateralized to the left hemisphere. This interpretation is supported by our further finding that atypical auditory responses in at-risk infants consistently predicted syntactic processing between 18 and 30 months and word production at 18 and 21 months of age. These results suggest a possible early marker of risk for dyslexia in at-risk infants.

## Highlights

• Auditory functioning was assessed in infants at risk for dyslexia using MEG.
• Atypical larger and longer neural responses in at-risk infants at 12 than 6 months.
• Responses were estimated to come from left temporal and left prefrontal regions.
• Neural responses predicted subsequent language skills evaluated at 18–30 months.
• These responses may serve as an early marker of risk for dyslexia.

## 1 Introduction

Individuals with developmental dyslexia commonly experience poor phonological processing skills, affecting their ability to recognize and manipulate the sound structure of words (Bradley and Bryant, 1978). It has been suggested that these deficits arise from atypical processing of basic auditory information, which was reported in children and adults with dyslexia (Goswami et al., 2011, Hämäläinen et al., 2013, Tallal, 1984). It is less clear whether poor basic auditory processing can already be identified early in development, with links to later language acquisition and reading skills. Dyslexia is strongly hereditary (Galaburda et al., 2006) and it is thus reasonable to assume that any potential deficits in basic sensory processing are detectable early in development. This allows investigations of the predictive power of auditory dysfunctions in infants (with follow-up language tests) before detectable symptoms of the condition emerge.

The current study examines basic auditory processing in infants at risk for dyslexia across the “sensitive” period for native-language phoneme perception (Kuhl, 2004, Peña et al., 2012). This period is characterized by a change in infants’ initial ability to perceive phonetic contrasts used to distinguish words across all languages at 6 months, to a narrowing of perception occurring by 12 months as infants’ speech perception abilities begin to specialize in phonetic units used only in the language(s) to which they are exposed (Kuhl et al., 2006, Werker and Lalonde, 1988).

Multiple studies suggest that perceptual narrowing is reflected in the brain as increased neural efficiency in both the temporal processing and the spatial distribution of the neural activation. Event-related potential (ERP) and MEG studies show a decrease of strength and latency of the neural signal to native speech contrasts and non-speech sound contrasts as typically developing (TD) infants age during their first year of life (EEG: Jing and Benasich, 2006, Kushnerenko et al., 2002, Ortiz-Mantilla et al., 2016; MEG: Bosseler et al. (2013)). These findings indicate that auditory processing in TD infants becomes more efficient with age. Also, studies of word learning in children demonstrate that earlier in development (13 months), children’s ERP responses to known compared to unknown words are broadly distributed across both hemispheres. With maturation and learning, responses to known words become more focal both spatially in the left hemisphere and temporally in terms of ERP width (Mills et al., 1997, Mills et al., 2005). An increase in neural efficiency in auditory perception has been further linked to faster reading speed in older children (7–8 years) (MEG work: Parviainen et al., 2011, Parviainen et al., 2019).

In contrast, risk for dyslexia manifests itself in slower and less efficient responses to auditory information. This has been documented in older children at risk for dyslexia (20-24 months) as indexed by delayed or absent neural ERP responses to linguistic material (Cantiani et al., 2017, Torkildsen et al., 2007) and early in development in infants at risk for dyslexia (newborn-6 months) demonstrating atypical neural ERP responses to both language (Guttorm et al., 2001, Guttorm et al., 2003, Leppänen et al., 1999, Leppänen et al., 2002, Pihko et al., 1999, Thiede et al., 2019, van Leeuwen et al., 2006) and basic auditory stimuli (Cantiani et al., 2016, Leppänen et al., 2010). Specifically, these infants showed deficits of processing auditory information at an early cortical stage when listening to tones (Leppänen et al., 2010) and phonemes presented with equal probability (Guttorm et al., 2001, Guttorm et al., 2003) and at a slightly later stage when discriminating speech sounds with changes in vowel identity, vowel and consonant duration, and syllable frequency (Leppänen et al., 1999, Leppänen et al., 2002, Pihko et al., 1999, Thiede et al., 2019, van Leeuwen et al., 2006) and tones with changes in frequency and duration (Cantiani et al., 2016, Leppänen et al., 2010). Infants’ ERPs were also distributed differently from a spatial perspective, such that at-risk newborns with later reading problems showed more left-lateralized than typical right-lateralized responses (Cantiani et al., 2016, Leppänen et al., 2010). Critically, auditory dysfunctions in early infancy/childhood are linked to poorer later language and literacy skills in school (Cantiani et al., 2016, Lohvansuu et al., 2018, Molfese, 2000, Schaadt et al., 2015, van Zuijen et al., 2013).

It remains undetermined whether at-risk infants continue to show auditory processing deficits across the sensitive period of native-language phoneme learning. If so, it may alter these infants’ neural processing of the auditory environment of the language(s) to which they are exposed. This could result in a variety of later symptoms such as poor native phoneme representations, and problems with language and reading skills.

The present study examined different samples of infants at risk for dyslexia and matched control infants at 6 and 12 months, when performance on native sound discrimination increases and performance on non-native sound discrimination declines (Kuhl et al., 2006, Werker and Lalonde, 1988). We examined auditory functioning at a basic processing level by repeatedly presenting white noise stimuli, which undergo comparatively less cortical processing than previously used tones (Cantiani et al., 2016, Leppänen et al., 2010) or more complex language sounds (Guttorm et al., 2001, Guttorm et al., 2003, Leppänen et al., 1999, Leppänen et al., 2002, Pihko et al., 1999, Thiede et al., 2019, van Leeuwen et al., 2006). In contrast to prior ERP work (Cantiani et al., 2016, Guttorm et al., 2001, Guttorm et al., 2003, Leppänen et al., 2010, Leppänen et al., 1999, Leppänen et al., 2002, Lohvansuu et al., 2018, Molfese, 2000, Pihko et al., 1999, Schaadt et al., 2015, Thiede et al., 2019, van Leeuwen et al., 2006, van Zuijen et al., 2013), which provides only limited information on the spatial distribution of neural activation, we used MEG for examining both temporal and spatial characteristics of neural source activation. MEG goes beyond what ERP studies can reveal because it permits reliable distinction between sources in the left and right auditory cortices and the separation of functionally distinct processes that may indicate different levels of maturation in the developing brain (Hämälainen et al., 1993).

Based on the research findings outlined above, we expected to find a maturational increase in neural efficiency in TD infants as indexed by temporally shorter/less strong and spatially more focal neural responses to white noise. In contrast, we expected that at-risk infants would start out with deficiencies in auditory processing and demonstrate continued aberrant neural efficiency by the end of the first year indicated by delayed and/or larger neural responses to white noise compared to the TD infant group. We further expected to find more pronounced deficiencies in the left hemisphere based on prior MEG research in children with specific language impairment SLI (suggested to share some genetic etiology with dyslexia) that reported atypical larger and longer-lasting auditory evoked responses only in left hemisphere (van Bijnen et al., 2019).

Finally, consistent with our position that auditory processing during this sensitive period is critical to language learning, we further hypothesized that atypical auditory processing in at-risk infants would predict functional outcomes of later language skills. This hypothesis follows on our previous work showing that infants’ early neural responses to language can predict later language (Kuhl et al., 2008). To test this, we correlated auditory processing with later non-linguistic communication, perceptive, expressive and syntactic language skills at 13–30 months of age because similar measures were found to predict later literacy skills (Duff et al., 2015, Scarborough, 1990b).

## 2 Materials and methods

### 2.1 Participants

A cross-sectional sample of 31 6-month-old and 48 12-month-old infants participated in this study, except for three infants visiting our laboratory at both ages. Infants were assigned to two groups: infants with familial risk of dyslexia and control infants. Data from seven 6-month-old and 17 12-month-old infants were rejected due to inability to tolerate the head position indicator coils (9), inability to localize the head position indicator coils in the MEG (6), failure to complete a sufficient 60 epochs during data collection (6), or failure to obtain a reliable dipole signal (3).

The final sample consisted of 24 6-month-old and 29 12-month-old infants. The 6-month-old group included 12 at-risk (5 males) and 12 control infants (5 males) and the 12-month-old group 14 at-risk (6 males) and 15 control infants (7 males). All infants were English learning, with English as the only language spoken at home, had no reported hearing difficulties, no history of ear infections, were born full-term (between 39 and 42 weeks of gestational age), and had typical birth weight between 6 and 10 lbs. We found no significant differences in mean age (6-month-old infants (one-way-ANOVA: *p* = .135): 190.83 ± 4.9 days (at risk), 193.75 ± 4.3 days (control); 12-month-old infants (*p* = .457): 373.1 ± 8.9 days (at-risk), 370.6 ± 8.7 days (control)) and gender between at-risk and control infants (Pearson’s chi-square: 6 months: *χ*^2^ (1) = 0.0, *p* = 1.0, 41.7% males; 12 months: *χ*^2^ (1) = 0.042, *p* = .837, 44.8% males). Written informed consent in accordance with the Human Subjects Division at the University of Washington was obtained from the parents.

### 2.2 Cognitive testing

Parents in the control group had no prior diagnosis of dyslexia or reading problems and no biological relative with dyslexia or reading problems. Parents with dyslexia had a prior diagnosis of dyslexia by a registered professional and a biological first-degree family member with a prior diagnosis of dyslexia or reading problems. Parents with a history of other learning difficulties or any type of language, speech or neurological disorder were excluded.

Differences in reading skills between control parents and the parent with dyslexia were examined with cognitive testing (62 out of 80 parents participated). Full Scale IQ-2 (FSIQ-2) and Verbal IQ (VCI) were measured with subtests vocabulary, matrix reasoning, and similarity of the Wechsler Abbreviated Scale of Intelligence® Second Edition (WASI-II) (Wechsler, 2011). Parents’ reading and spelling abilities were assessed with letter-word identification (LW), passage comprehension (PC), and word attack (WA), and spelling of Woodcock Johnson® IV (WJ IV) Tests of Achievement Form A (Schrank et al., 2014a). Subtests LW and WA were timed to assess the speed of single word and pseudoword reading. Long-term retrieval was assessed with subtests story recall and visual-auditory learning of the WJ IV Tests of Cognitive Abilities (Schrank et al., 2014b).

### 2.3 Socioeconomic status (SES)

The groups were matched on SES as measured with the widely used Hollingshead scale index (Hollingshead, 1975). 46 families out of 53 families in total (86.8%) completed the SES questionnaire. The Hollingshead scale produces an index between 8 and 66 indicating parental education and occupation, with higher values corresponding to higher SES. Families of six-month-olds had a mean Hollingshead index and SD of 52.95 ± 9.6 for infants at risk for dyslexia and 52.6 ± 5.7 for control infants. Families of twelve-month-olds showed a mean and SD of 55 ± 10.8 for infants at risk for dyslexia and 57.3 ± 6.4 for control infants. A two-way ANOVA examining the effects of Group and Age on SES yielded no significant differences in SES between the families of at-risk and control infants (*p* = .7) and between the families of 6-month-old and 12-month-old infants (*p* = .169) suggesting that results obtained in this study cannot be accounted for by differences in SES.

### 2.4 Stimuli

**Fig. 1:**
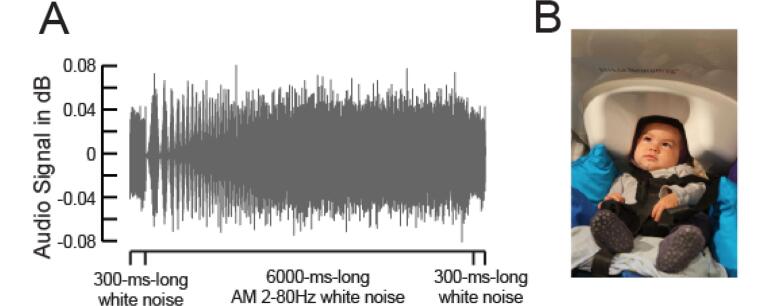
*(A)* Acoustic waveform of white noise stimulus. *(B)* Example of infant under the MEG helmet during recording.

Acoustic stimuli consisted of 300 ms white noise burst + 6000 ms amplitude-modulated (AM) white noise + another 300 ms white noise burst (6.6 sec long in total) with a constant envelope and a randomized white-noise carrier to diminish habituation effects of the signal throughout the experiment (Fig. 1a). The sound modulation rate linearly increased from 2 to 80 Hz in 6 sec and there were 110 different variants of the stimulus (random white noise). Stimuli were presented with a silent interstimulus interval that varied between 1000 and 1200 ms at 65 dB SPL through loudspeakers using Expyfun Version 2.0. The motivation for the stimulus choice was to investigate temporal sampling of auditory information. Results concerning the responses to the AM part of the stimulus will be reported in a subsequent manuscript.

### 2.5 MEG recording

MEG data were recorded in a magnetically shielded room with a whole head adult-sized 306 channel Elekta Neuromag® MEG system (Elekta Oy, Helsinki, Finland). Using Fastrak® 3D digitizer (Polhemus, Colchester, VT, USA), we digitized three anatomical landmarks (left and right preauricular points, nasion) to construct an individual Cartesian head-centric coordinate system, five HPI coils and about 100 additional points along the head surface. Data collection began when infants were seated calmly in a custom-made chair under the MEG helmet (Fig. 1b). Research assistants entertained infants with silent toys while a silent video of baby faces was played in the background. MEG data were recorded with an analog band-pass filter of 0.03–330 Hz and a sampling rate of 1.2 kHz. Positions of infants’ heads relative to the sensor array were tracked continuously by extracting the magnetic fields emitted by HPI coils at frequencies between 83 and 323 Hz. Any channels with amplitudes below a certain level were considered ‘flat’, removed (grad = 1e-13, mag = 1e-15), and reconstructed with the signal space separation (SSS) method (Taulu et al., 2005) during preprocessing.

### 2.6 MEG analysis

MEG data were preprocessed using MNE-Python (version 0.11.0) (Gramfort et al., 2013, Gramfort et al., 2014). Data were processed using Maxwell Filter (Gramfort et al., 2014) to apply temporal SSS (tSSS) (Taulu and Hari, 2009). After tSSS, movement compensation (Larson and Taulu, 2017) was applied (MaxFilter™ software, Elekta Neuromag®, Elekta Oy, Helsinki, Finland) and transformed to the median of each individual’s head position to minimize reconstruction noise. tSSS was performed in 8-sec time windows with a correlation limit of 0.95 for environmental noise reduction. Automatic cardiac suppression with signal space projection was applied with two magnetometer and two gradiometer projectors.

For sensor level and ECD modeling, data of all infants were transformed to the same head position (mean position across all infants) using movement compensation (two-way ANOVAs of the median absolute deviation of 1) head position and 2) head angle showed no significant main effects; *p* > 0.05, all) and further band-pass filtered (1–80 Hz). Averaged epochs of 8000 ms after each stimulus onset, including a 200-ms prestimulus period, were baseline corrected with respect to the mean amplitude of the prestimulus period, and downsampled to 200 Hz. Location, strength, and orientation of neural sources were estimated with ECDs in a spherically symmetric head model. The ECDs were fitted every 5 ms and the best dipole was chosen for further analysis. One dipole per hemisphere for each infant was fitted around the peak of an early deflection at 100 to 250 ms using a pre-selected template of approximately 30 MEG channel triplets (Elekta Neuromag Source Modeling® software). Only ECDs explaining ≥ 70% of the measured field (goodness-of-fit, GoF) were included in further analysis.

We observed a large difference in a deflection of individual dipole moments between 100 and 500 ms after stimulus onset between the groups (i.e., likely encompassing most of the onset, sustained, and offset responses to the burst of the three-part white noise stimulus; see Results, Fig. 3a). Potential differences between groups (at-risk infants, control infants) and ages (6 months, 12 months) at the deflection were examined using two measures: mean activations (MAs) and duration responses of the auditory dipole responses. MAs of dipole responses were calculated by averaging the dipole moments with GoF ≥ 70% within 100 to 500 ms. For statistical analysis, MAs were log-transformed to obtain approximately normally distributed data. The duration of the dipole responses was calculated as the time span for which the dipole activation was contiguously within 50% of the maximum peak activation between 100 and 500 ms. Effects of Group and Age on MAs and the duration of the auditory dipole responses were examined with a two-way analysis of variance (ANOVA) separately for the left and right hemisphere.

For distributed source estimates (noise-normalized minimum-norm estimates), a noise covariance matrix was calculated from -200 ms to 0 ms before stimulus onset. A 14-month-old template brain was used as anatomical model for localization, consisting of a 1-layer boundary element model surface (conductivity = 0.3 S/m) and a surface source space consisting of 4098 dipoles per hemisphere placed uniformly along the gray-white matter boundary defined using FreeSurfer (Dale et al., 1999). The template surfaces were deformed to create a surrogate head model for each subject individually using an affine transformation (3-dimensional scaling, rotation, and translation) of the template to minimize the distance between the surrogate outer scalp surface and the true digitized head shape of the subject (resulting co-registration error: M = 3.23, SD = 2.01 mm). This surrogate head model was used to compute a forward model for the data (linear collocation approach; Hämäläinen et al., 1993), which was combined with the baseline noise covariance to compute an anatomically constrained dSPM inverse operator (default depth weighting exponent 0.8 and λ^2^ = 1/9 were used; Dale et al., 2000). Because of the potential mismatch between the cortical folding of the individual subject and the surrogate, free source orientations (not constrained to be normal to cortex) were used. This operator yielded values proportional to F-statistics based on activation levels relative to the baseline period in three cardinal directions for each cortical vertex. The norm across directions for each vertex was taken to obtain a total activation level for each vertex as a function of time, and these were then averaged across 100 to 500 ms to obtain a spatial activation map for each subject. We examined three contrasts that reflect the three terms effectively computed in a standard two-way ANOVA: the main effect terms (Age: 6 months vs. 12 months; and Group: control vs. at-risk) and the interaction term (Group X Age: [6-month control plus 12-month at risk] vs. [6-month at risk plus 12-month control]). Based on the ECD results, we had an a priori hypothesis about the Group X Age interaction term, which we spatially explored by contrasting two groups of values, the first comprised of those from 6-month-old control and 12-month-old at-risk infants, and the second of those from 12-month-old control and 6-month-old at-risk infants. These interaction effects were tested while controlling for multiple comparisons via a whole-brain non-parametric spatial clustering permutation test with a maximal statistic (using conservative control of the family-wise error rate), testing the null hypothesis that the data from the two groups (here, the 6-month control plus 12-month at-risk vs. 6-month at-risk plus 12-month control) came from the same underlying distribution. The clustering algorithm operated by clustering vertices within each permutation that survived a *p* < .05 (uncorrected) threshold, with vertices clustered based on geodesic spatial proximity (Larson and Lee, 2013).

### 2.7 Language abilities

Receptive and expressive aspects of language and nonverbal communication were measured at 13 and 15 months with subsections words understood, words produced and gestures (early, late and total amount) of the infant form of the MacArthur-Bates Communicative Development Inventories (CDI, infant form: words and gestures) (Fenson et al., 1993). Expressive and syntactic language skills were assessed at 18, 21, 24, 27 and 30 months with subsections on words produced, M3L, and grammatical complexity of the toddler form of the CDI. Parents were asked to complete CDI forms on the day their child reached the target age. Data from one at-risk child at 13, 15 and 18 months and two control children at 13 and 15 months were missing because parents were unable to complete the CDI. Also, data from one control child was removed from analysis because M3L scores at 21, 24, and 27 months were larger than 3 SDs above the mean of M3L scores in that group.

The Pearson product-moment correlation coefficient was used to examine a possible association between MAs and dipole durations in the left and right hemisphere and percentile of words understood, words produced, early gestures, late gestures and total amount of gestures at 13 and 15 months and percentile of words produced, M3L, and percentile of sentence complexity at 18, 21, 24, 27 and 30 months. MAs were collapsed across age to increase statistical power.

## 3 Results

Parents with dyslexia and control parents did not significantly differ in age, gender, and FSIQ-2 (Supplementary Material, Table S1). Parents with dyslexia had significantly poorer yet above average VCIs and significantly differed from control parents in reading (LW, PC), basic reading (LW, WA), reading speed for words and pseudowords, spelling, and long term-retrieval.

**Fig. 2:**
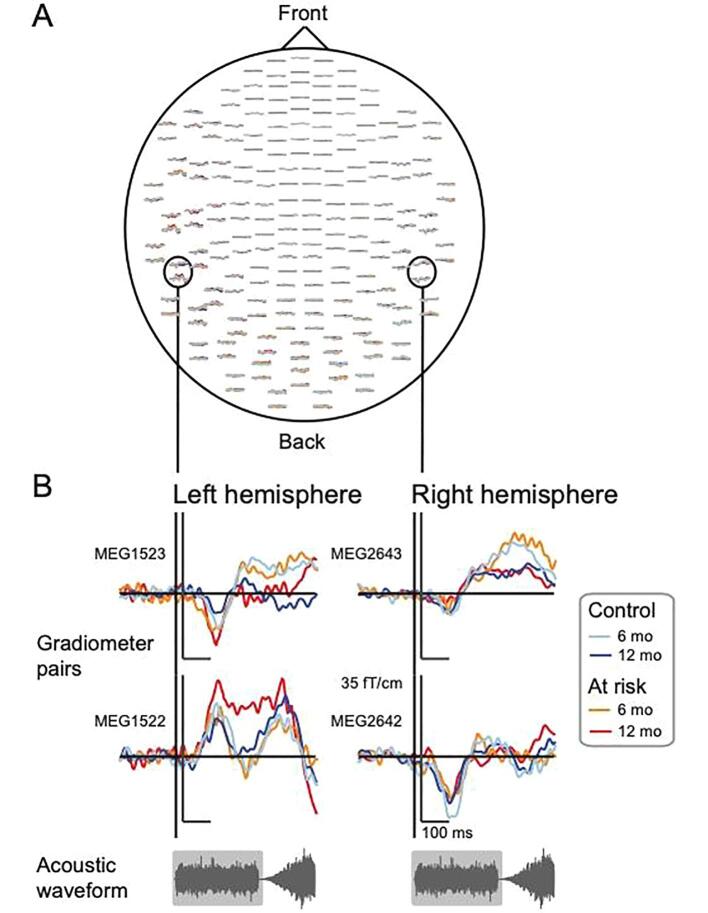
*(A)* Grand-averaged magnetic responses to white noise for 6- and 12-month-old at-risk and control infants at 204 planar gradiometers. *(B)* Example grand-averaged magnetic responses to white noise (300-ms long white noise burst highlighted in gray at bottom) from two example gradiometer pairs positioned over the left and right temporal regions.

Fig. 2a shows reliable grand-averaged sensor-level waveforms with high signal-to-noise ratio in response to white noise. By visual inspection, the upper sensor in the gradiometer pair over both hemispheres indicates decreased amplitude by age, and the lower sensor in the gradiometer pair over the left hemisphere showed an increase by age for the at-risk infants (Fig. 2b).

**Fig. 3:**
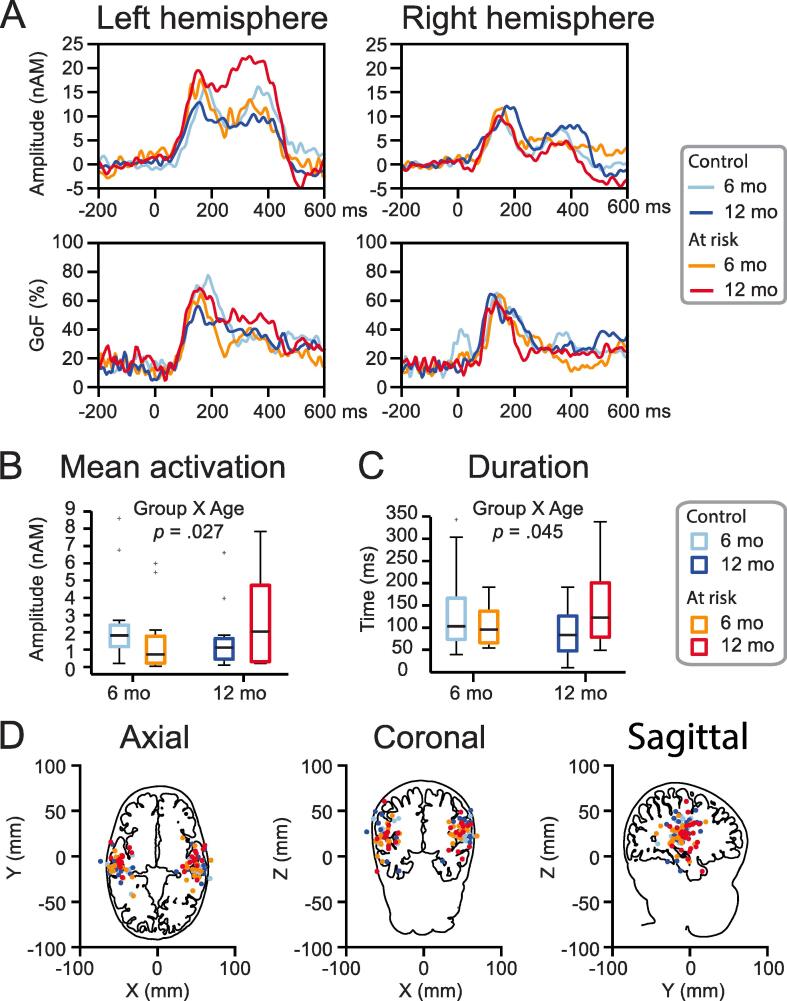
(A) Grand-average dipole moment waveforms and goodness of fit (GoF) (%). (B) Box and whisker plots show median, and the bottom and top edges of the box indicate the 25th and 75th percentiles, respectively. Outliers (more than 1.5 times the interquartile range) are displayed with a grey + sign. Group X Age interaction effect in the left hemisphere for MA: Larger MAs in control than at-risk infants at 6 months, whereas relationship was reversed at 12 months. (C) Similar pattern for dipole durations: longer durations in control than at-risk infants at 6 months, whereas pattern was reversed at 12 months. *(D)* Locations of dipole moments.

Potential differences between groups (at-risk infants, control infants) and ages (6 months, 12 months) were examined at the source level in the brain by individually fitting equivalent current dipoles (ECDs) in the left and right auditory cortices. We examined group and age differences at a deflection of individual dipole moments between 100 and 500 ms after stimulus onset between the groups (i.e., likely encompassing most of the onset, sustained, and offset responses to the burst of the three-part white noise stimulus; Fig. 3a). A two-way analysis of variance (ANOVA) on log-transformed mean activations (MA) of dipole responses between 100 and 500 ms yielded a significant Group X Age interaction (*F* (1,49) = 5.196, *p* = .027, partial eta squared *η_p_^2^* = 0.096) in the left hemisphere. This significance was not observed for the untransformed mean activation data (*p* = .068). Mean, SD, and *p*-values are reported for both log-transformed and untransformed data, with the former ones marked with an asterisk sign* in the remaining paragraph. The significant interaction term implies that the MA measure in the control infants at 6 months and the at-risk infants at 12 months (M = 5.07*, SD = 1.16 nAm*, M = 2.67, SD = 2.53 nAm) was significantly different from the control infants at 12 months and the at-risk infants at 6 months (M = 4.27*, SD = 1.38 nAm*, M = 1.49, SD = 1.84 nAm) (Fig. 3b). However, interestingly, breaking this interaction down into individual contrasts, none were significant: at-risk infants vs. control infants at 6 months (*p* = .072*, *p* = .309), at-risk infants vs. control infants at 12 months (*p* = .213*, *p* = .113), at-risk infants at 6 months vs. at-risk infants at 12 months (*p* = .133*, *p* = .196), and control infants at 6 months vs. control infants at 12 months (*p* = .098*, *p* = .202). The significant interaction term thus was driven by the combination of the higher values for the control infants at 6 months (M = 5.14*, SD = 0.97 nAm*, M = 2.52, SD = 2.53 nAm) and the at-risk infants at 12 months (M = 5.01*, SD = 1.34 nAm*, M = 2.79, SD = 2.62 nAm) compared to the control infants at 12 months (M = 4.41*, SD = 1.17 nAm*, M = 1.45, SD = 1.73 nAm) and the at-risk infants at 6 months (M = 4.1*, SD = 1.63 nAm*, M = 1.55, SD = 2.1 nAm). The main effect for both Group (*p* = .541*, *p* = .771) and Age (*p* = .802*, *p* = .893) rendered non-significant. This pattern was not observed in the right hemisphere (*p* = .843*, *p* = .667).

**Fig. 4:**
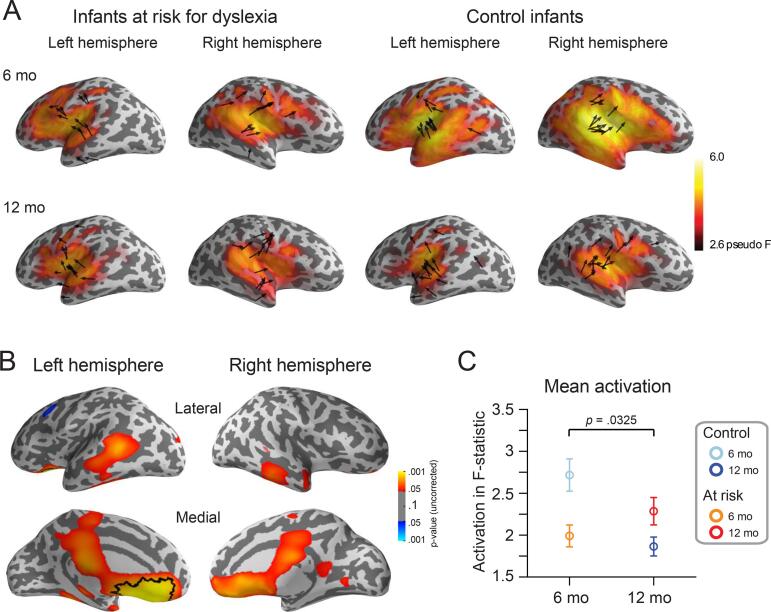
*(A)* Location and orientation of ECDs (arrows) superimposed on the grand-averaged (across subjects) noise-normalized activation (dSPM) maps for 6- and 12-month-old at-risk and control infants for left and right hemisphere. (*B*) Statistical 2-tailed *t*-test maps show Group X Age interaction (i.e., a contrast of [6-month control and 12-month at risk] vs. [6-month at risk and 12-month control]) in left temporal (lateral view) and left and right frontal regions (medial view). When controlling for multiple comparisons, a significant interaction effect emerged based on a left frontal region (*p* = .034, black contour line). (*C*) Interaction effect in the left frontal region from (B). Error bars indicate standard error of the means.

For the duration of the auditory dipole responses, there was a significant Group X Age interaction in the left hemisphere (two-way ANOVA: *F* (1,49) = 4.243, *p* = .045, *η_p_^2^* = 0.080). The significant interaction term implies that the duration measure in the control infants at 6 months and the at-risk infants at 12 months (M = 142.48, SD = 93.8 ms) was significantly different from the control infants at 12 months and the at-risk infants at 6 months (M = 98.36, SD = 48.71 ms) (Fig. 3c). When breaking this interaction down into individual contrasts, none were significant: at-risk infants vs. control infants at 6 months (*p* = .337), at-risk infants vs. control infants at 12 months (*p* = .058), at-risk infants at 6 months vs. at-risk infants at 12 months (*p* = .147), and control infants at 6 months vs. control infants at 12 months (*p* = .170). The significant interaction term thus was driven by the combination of the higher values for the control infants at 6 months (M = 134.75 ms, SD = 96.94 ms) and the at-risk infants at 12 months (M = 149.1 ms, SD = 94.17 ms) compared to the control infants at 12 months (M = 93.43 ms, SD = 53.02 ms) and the at-risk infants at 6 months (M = 104.53 ms, SD = 44.24 ms). The main effect for both Group (*p* = .544) and Age (*p* = .938) rendered non-significant. This pattern was not observed in the right hemisphere (*p* = .594). No effects were found for locations (*p* = .070 - 0.857) and orientations (*p* = .073 - 0.916) of the ECDs between Group and Age in the left and right hemisphere (Fig. 3d and 4a).

We examined effects on the spatiotemporal source distributions of the entire cortex with dynamic statistical parametric mapping (dSPM) (Dale et al., 2000), performing analysis on time-averaged (100 to 500 ms) dSPM activation magnitudes in source space (Fig. 4a). Consistent with the ECD results, in the uncorrected statistical maps there was a Group X Age interaction in the left temporal region driven by larger activation in control than at-risk infants at 6 months, whereas the pattern was reversed at 12 months (Fig. 4b, lateral view). This effect was also more pronounced in the left than right frontal regions (Fig. 4b, medial view). When controlling for multiple comparisons using spatial clustering with a maximal statistic across the entire cortical surfaces, the interaction effect in the left frontal region allowed us to reject the null hypothesis (*p* = .0325, Cohen’s d = 1.01; Fig. 4c). According to a cortical parcellation derived from Human Connectome Project data (Glasser et al., 2016), this left frontal region consists of vertices predominantly in the orbital and polar frontal cortex (36% of vertices) and in the anterior cingulate and medial prefrontal cortex (25% of vertices).

**Fig. 5:**
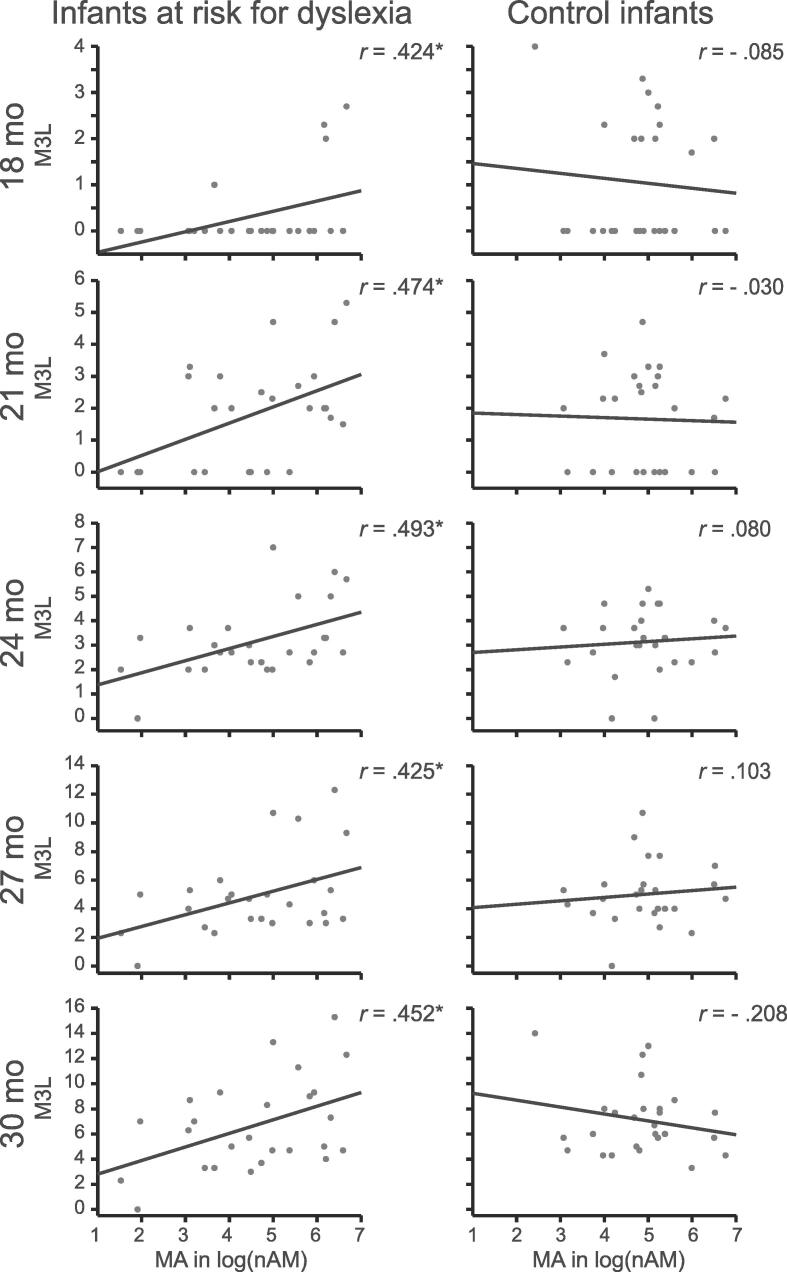
Scatterplots show correlations between infants’ MA in the left hemisphere and their syntactic abilities at 18, 21, 24, 27 and 30 months. Syntactic skills were more pronounced in control than at-risk children at 18 months. Atypical MAs consistently predicted syntactic abilities at 18 to 30 months in at-risk (left column; *p < .05) but not in control children (right column).

We investigated receptive and expressive aspects of language and non-linguistic communication skills in the same children at 13 and 15 months (results are reported in Supplementary Material, Table S2) and syntactic proficiency assessing the mean length of morphemes in the three longest utterances (M3L) and grammatical complexity, and vocabulary production at 18, 21, 24, 27 and 30 months. We found the most consistent results for the M3L measure. Syntactic development as indexed by M3L (Scarborough, 1990a) was more pronounced at earlier ages in control than at-risk children as 11 control compared to four at-risk children already produced complex syntactic structures at 18 months (Fig. 5, top panel). MAs in at-risk infants significantly correlated with M3L at 18, 21, 24, 27 and 30 months. Atypical MAs in the left hemisphere in at-risk infants, collapsed across 6 and 12 months, consistently predicted M3L at 18 months of age (Pearson *r* = 0.424, *n* = 25, *p* = .035), 21 months (*r* = 0.474, *n* = 26, *p* = .014), 24 months (*r* = 0.493, *n* = 26, *p* = .011), 27 months (*r* = 0.425, *n* = 26, *p* = .03), and 30 months (*r* = 0.452, *n* = 26, *p* = .02) (Fig. 5, left column) with larger MAs linked to greater M3L at 18 to 30 months of age. In contrast, all correlations between left MAs and later M3L were non-significant in control infants (*p* = .307 - 0.885) (Fig. 5, right column). We found no significant correlations for MAs in the right hemisphere and later M3L in at-risk (*p* = .144 - 0.92) and control infants (*p* = .332 - 0.991). Correlations for the duration of the auditory responses and later M3L were also not significant in either group (at-risk: *p* = .248 - 0.624, control: *p* = .114 - 0.904 (left hemisphere); at-risk: *p* = .521 - 0.920, control: *p* = .387 - 0.795 (right hemisphere)), indicating that this measure was not a reliable predictor for later M3L. Similar correlation results emerged for grammatical complexity and vocabulary production, though less consistent over time and therefore, are reported in Supplementary Material, Fig. S1.

## 4 Discussion

This study is the first to examine low-level auditory processing in infants at risk for dyslexia across the sensitive period for native phoneme learning when infants transition from detecting phoneme contrasts of all languages to becoming language-bound listeners (Kuhl, 2004). We found atypical early stimulus-driven magnetic responses to white noise in at-risk infants, as reflected by a group difference in response pattern across time. This effect was significant both when fitting ECDs and when using distributed source modeling and localized to the same cortical areas regardless of the modeling parameters/algorithms used to estimate the current distribution underlying the measured activity. Its localization to the left temporal and left frontal regions indicated its relevance on later language abilities which was confirmed by our further finding of a strong link between left-hemisphere brain responses in at-risk infants and their later syntactic proficiency beginning from 18 through 30 months of age.

The interaction term for mean activations and duration responses in the auditory cortex suggested that the trend goes in opposite directions for the two groups. Consistent with our hypotheses, activations in the auditory cortex tended to be smaller and shorter in the left hemisphere with increasing age in TD infants relative to age-matched at-risk infants suggesting that TD infants’ brains become more efficient in processing simple sounds between 6 and 12 months of age. Previous research with TD infants found similar results of a decrease of ERP amplitudes and latencies with increasing age for tone (Jing and Benasich, 2006, Kushnerenko et al., 2002) and word contrasts (Mills et al., 1997, Mills et al., 2005) and interpreted this pattern as an increase in neural efficiency as a function of learning – an interpretation consistent with our data.

At-risk infants did not show a pattern of increased neural efficiency over time, but instead a shift to atypical larger and longer activations to simple sounds in the auditory cortex. This was especially pronounced in the left hemisphere – a result that agrees with prior research in children with SLI showing atypical larger and longer-lasting auditory evoked responses only in the left hemisphere (van Bijnen et al., 2019). Also, auditory processing in at-risk infants was compromised early in time (starting at 100 ms after stimulus onset, Fig. 2b), similar to previous findings in infants at risk for dyslexia at birth and 6 months (ERPs to non-linguistic simple tones) (Cantiani et al., 2016, Leppänen et al., 2010) and children with language development problems (ERPs at 100 ms to words) (Mills et al., 2005). This suggests that auditory processing in at-risk infants is impaired at lower levels of auditory information processing with potential consequences for higher-order cognitive processes such as the generation of abstract spectro-temporal categories (e.g., phoneme representations). Our findings show that risk for dyslexia manifests itself in early detectable basic auditory processing deficits across the critical period of phoneme learning.

Functional neuroimaging and E/MEG studies yielded differences in brain function and connectivity that are markers for dyslexia (Gaab et al., 2007, Gabrieli, 2009, Hämäläinen et al., 2015, Helenius et al., 2002, Hoeft et al., 2007, Khan et al., 2011, Shaywitz et al., 2002, Temple et al., 2003). When performing phonological tasks, TD readers recruit multiple brain areas including the left temporal region (Shaywitz et al., 2002, Temple et al., 2003). In contrast, data from children and adults with dyslexia found underactivation in the left temporal region (Gaab et al., 2007, Hämäläinen et al., 2015, Helenius et al., 2002, Hoeft et al., 2007, Khan et al., 2011, Shaywitz et al., 2002, Temple et al., 2003). This underactivation is evident when children with dyslexia are compared to reading-level matched controls and was therefore regarded as fundamental to dyslexia per se rather than related to delayed maturation or reading level (Hoeft et al., 2007). Our results of atypical activation in the left temporal region for simple sounds agree with this reasoning. Furthermore, whereas prior research linked left temporal deficits primarily to phonological processing problems (Hoeft et al., 2007, Temple et al., 2003), our findings suggest that risk for dyslexia involves a general impairment of this region for processing acoustic stimuli unrelated to language. This is supported by studies in TD and SLI children showing a link between less efficient neural sound processing and poorer reading speed (Parviainen et al., 2011, van Bijnen et al., 2019). Worth noting is that we observed over activation in 12-month-old at-risk infants in contrast to prior findings of under activation in this region in children and adults with dyslexia. Inconsistencies in the direction of atypical processing could likely stem from ongoing developmental changes in brain networks, as was recently shown for oscillatory networks underlying native phoneme processing when compared between 12-month-olds and adults (Bosseler et al., 2013).

Based on the interaction analysis (Fig. 4b), auditory processing was also altered in left frontal areas in at-risk infants, a finding that is consistent with data of children and adults with dyslexia showing abnormal activity in this region for auditory processing that is important for language and reading (phonological processing and verbal working memory tasks) (Gaab et al., 2007, Hoeft et al., 2007, Shaywitz et al., 2002, Temple et al., 2003). Deficits in frontal areas were linked to later reading skills and not dyslexia itself and interpreted as compensatory mechanisms for a failure to accurately develop a temporal posterior reading system (Hoeft et al., 2007, Temple et al., 2003). Unlike these studies, we measured at-risk infants with a passive listening paradigm including simple sounds unrelated to language suggesting that risk for dyslexia entails a rather fundamental deficit in left frontal regions for processing simple sounds. In addition, these studies examined alphabetic readers, whereas studies in Chinese readers with dyslexia interpreted deficits in left frontal regions as fundamental markers for dyslexia suggesting that experience shapes cognitive strategies and in turn, tunes the cortex for reading development (Siok et al., 2004). Our infant data show that aberrant left frontal processing occurs before native phoneme skills and mapping phonemes with visual information such as graphemes or characters are acquired. An alternative explanation for this frontal effect could be differences in attention between at-risk and control infants. Anterior cingulate, specifically, has previously been linked to top-down attentional control (Bush et al., 2000), and therefore it is possible that risk for dyslexia is involved in atypical self-regulation of attention for processing non-linguistic sounds. The difference in picking up frontal activity between our focal and distributed models could be because frontal activity is not as easily representable by our single-dipole model. In addition, the channel selection that we used in dipole fitting was not favorable for frontal areas while there were no restrictions on the channels applied in the analysis of distributed activity.

Our study identified poorer syntactic skills in at-risk children already at 18 months – at a time in which children are just beginning to tap into the nature of syntactic rule systems. This is 1 year earlier than previously reported (Scarborough, 1990b). Our study also established that less efficient low-level auditory processing in the left hemisphere consistently predicts syntactic skills from 18 through 30 months in at-risk children. This is consistent with prior work demonstrating that larger and longer-lasting auditory responses in the left hemisphere in TD and SLI children (9-10 years) predicted later poorer reading speed (Parviainen et al., 2011, van Bijnen et al., 2019), emphasizing the role of auditory processing skills as an early marker in the developmental trajectory of dyslexia, followed by phonemic awareness, syntactic skills, letter-sound knowledge, and reading skills (Scarborough, 1990b). A link between low-level auditory processing and later syntactic proficiency is not surprising because both processes involve aspects of learning to extract regularities (Mueller et al., 2012). By presenting the same sounds repeatedly, our paradigm likely elicited mechanisms of regularity detection. Deficits in low-level auditory processing can be interpreted as resulting from deficiencies in these mechanisms, consistent with previous studies in newborns that later developed dyslexia (Leppänen et al., 2010) and in adults with dyslexia (Ahissar, 2007) and in line with notions suggesting that individuals with dyslexia have a general difficulty in extracting stimulus regularities from auditory inputs (Ahissar et al., 2006). Interestingly, greater responsiveness to repeated acoustic material in at-risk infants was beneficial for their later syntactic regularity production. This finding likely reflects early compensatory mechanisms which may involve the recruitment of a larger set of neurons (Mills et al., 2005) or a possible morphological enlargement of some regions in the auditory cortex (Serrallach et al., 2016). In contrast, prior work in TD children showed that lesser neural activation is linked to later improved reading skills, and the decrease of activation for auditory processing being indicative of more automatized neural processing (Albrecht et al., 2000, Parviainen et al., 2011).

MA was not a consistent predictor for syntactic abilities in all at-risk infants, as can be seen in the CDI data at 18 and 24 months, and additional measures should be explored in future research. Even though some at-risk infants may show a similar pattern to control infants, they still may exhibit later poorer reading skills. This was pointed out by longitudinal studies, such as Lyytinen et al. (2005) showing that many children who are at familial risk for dyslexia do not show delays in early language, but still face later problems in reading and/or spelling. Thus, it is noteworthy that even age-appropriate early language skills do not ensure norm-level fluent reading skills in at-risk children. Future studies may benefit from recruiting a larger number of subjects to delineate possible subgroups of infants at risk for dyslexia. Given these considerations, any discussion of causation, such as impaired low-level auditory processing results in later language perception and processing issues, should be treated with caution. Increased MAs in at-risk infants can be a consequence with larger amplitudes compensating for some problems in auditory or language-related functions. It is also possible that processing differences in the left hemisphere cause problems in such functions, or auditory and language deficits both mark an underlying neurodevelopmental disorder.

In order to move closer to causation, future research could examine younger infants at risk for dyslexia, perhaps newborns, and test these infants with very simple sounds, such as clicks that evoke auditory brainstem responses. By doing so, one would be able to pinpoint where exactly in the auditory pathways (from auditory nerve fibers to lateral lemniscus) auditory processing problems could occur. This can be done with MEG, as a previous study from our laboratory has successfully shown (Zhao and Kuhl, 2018). However, such an experimental set up may not be sufficient to address this question, because prior research by Partanen et al. (2013) showed that learning induced changes that are relevant for later speech processing can already occur in the womb.

Our study has two possible weaknesses. First, it would be optimal to obtain individual MRI’s from each subject as templates for source modelling. However, due to practical limitations with infant data this was not possible for the study at hand. There are several reasons why we have confidence in the 14-month-template used here. We previously successfully used the 14-month-template brain in studies including those with 11-month-olds (Ferjan Ramirez et al., 2017) and 9-month-olds (Zhao and Kuhl, 2016) and do not expect that the larger difference in age between the surrogate and the 6- vs. 12-month cohorts causes any problematic systematic bias here. The differences in scaling factors used to deform the surrogate MRI are relatively small. There is a related effect in the ECD modeling, which does not use the surrogate model. And, if there is some bias introduced, it should manifest as a main effect of age rather than an interaction term in the comparisons, so it is unlikely that this has affected our primary results. Secondly, we wish to note that sex can affect dyslexia differently (Krafnick and Evans, 2018). Because of the number of subjects in each group, we decided against including sex as a variable and aim to consider this as an improvement for future studies.

## 5 Data availability statement

All relevant data supporting the findings of this study are stored on a server at the Institute for Learning & Brain Sciences at the University of Washington and are available for research purposes on request by contacting P.K.K.

## Declaration of Competing Interest

The authors declare that they have no known competing financial interests or personal relationships that could have appeared to influence the work reported in this paper.
