# Supplementary material for: Auditory deficits in infants at risk for dyslexia during a linguistic sensitive period predict future language
Source: Neuroimage Clin. 2021 Feb 1;30:102578. doi: 10.1016/j.nicl.2021.102578 (PMC7892990; doi:10.1016/j.nicl.2021.102578)
Supplement: Supplementary data 1 [file mmc1.docx]

**Supplementary Material**

Fig. S1. *(A)* Scatterplots show correlations between infants’ MAs in the left hemisphere collapsed across 6 and 12 months and their syntactic language skills assessed with grammatical complexity at 21 and 30 months. Larger MAs in the left hemisphere in at-risk infants were associated with greater percentile scores in grammatical complexity at 21 (*r* = .526, *n* = 26, *p* = .006) and 30 months (*r* = .428, *n* = 26, *p* = .029) (*p < .05, left column), whereas correlations were non-significant in control infants (21 months: *r* = -.101, *n* = 26, *p* = .726) and 30 months: *r* = .197, *n* = 26, *p* = .335; right column). A similar response pattern emerged for correlations between dipole durations in the left hemisphere and grammatical complexity at 21 months (at-risk infants: *r* = .424, *n* = 26, *p* = .031; control infants: *r* = .03, *n* = 26, *p* = .884) and 27 months (at-risk infants: *r* = .464, *n* = 26, *p* = .017; control infants: *r* = -.086, *n* = 26, *p* = .677) *(B)*, and correlations between MAs in the left hemisphere and words produced at 18 months (at-risk infants: *r* = .426, *n* = 25, *p* = .034; control infants: *r* = -.162, *n* = 26, *p* = .43) and 21 months (at-risk infants: *r* = .458, *n* = 26, *p* = .018, control infants: *r* = -.132, *n* = 26, *p* = .52)) *(C)*.

Table S1. Cognitive test results for parents of 6- and 12-month-old infants

|  | | Parent with dyslexia | Control parents | *F* | *p* | *η_p_^2^* |
| --- | --- | --- | --- | --- | --- | --- |
| Parents of 6-month-old infants (8 parents with dyslexia, 22 control parents) | | | | | | |
| Age | | 33.75 (5)^a^ | 32.86 (5.4) | .164^b^ | .689 | .006 |
| Sex ratio: male/female | | 5/3 | 11/11 | *χ*^2^(1) = .368^c^ | .544 |  |
| FSIQ-2^d^ | | 114.88 (13.7) | 118.95 (12.4) | .601^b^ | .445 | .021 |
| VCI^d^ | | 109.88 (11.9) | 119.77 (11.5) | 4.270^b^ (4.564)^e^ | .048 (.042)^e^ | .132 (.145)^e^ |
| Reading^d^ | | 96.5 (7.7) | 110.59 (10.2) | 12.616^b^ (17.257) | .001 (.000) | .311 (.390) |
| Basic Reading^d^ | | 93.5 (12.5) | 110 (11.6) | 11.384^b^ (14.484) | .002 (.001) | .289 (.349) |
| Reading speed (words) | | 169.3 (186.4)^f^ | 86 (23.7) | 4.459^b^ (3.654) | .044 (.067) | .137 (.119) |
| Reading speed (pseudo words) | | 116.98 (101.3)^f^ | 71.59 (18.2) | 4.296^b^ (3.506) | .048 (.072) | .133 (.115) |
| Spelling | | 45.4 (28.3)^g^ | 70.5 (21)^g^ | 6.998^b^ (6.298) | .013 (.018) | .200 (.189) |
| Long-term Retrieval^d^ | | 107.4 (13.8) | 117.59 (10.4) | 4.755^b^ (3.931) | .038 (.058) | .145 (.127) |
| Parents of 12-month-old infants (10 parents with dyslexia, 22 control parents) | | | | | | |
| Age | 33.3 (5.7) | | 35.86 (3.4) | 2.545^h^ | .121 | .078 |
| Sex ratio: male/female | 3/7 | | 10/12 | *χ*^2^(1) = .681 | .409 |  |
| FSIQ-2 | 112.3 (8.8) | | 120.5 (12.4) | 3.594 ^h^ | .068 | .107 |
| VCI | 110.2 (9.7) | | 119 (10.8) | 4.915^h^ (1.220) | .034 (.278) | .141 (.040) |
| Reading | 99.3 (6.9) | | 111.2 (9.6) | 12.383^h^ (7.783) | .001 (.009) | .292 (.212) |
| Basic reading | 92 (9.1) | | 109.8 (10.5) | 21.417^h^ (16.493) | .000 (.000) | .417 (.363) |
| Reading speed (words) | 127.3 (37.9) | | 79.4 (21.2) | 21.172^h^ (16.469) | .000 (.000) | .414 (.362) |
| Reading speed (pseudo words) | 108.1 (21.2) | | 69.64 (19.1) | 25.987^h^ (20.074) | .000 (.000) | .464 (.409) |
| Spelling | 40.6 (17.9) | | 73.32 (16.06) | 26.571^h^ (20.215) | .000 (.000) | .470 (.411) |
| Long-term retrieval | 107 (14.8) | | 117.91 (11) | 5.727^h^ (3.047) | .023 (.091) | .160 (.095) |

^a^ Standard deviations are given in parentheses

^b^ *F*(1,29)

^c^ Pearson’s chi-square test

^d^ Standard scores

^e^ ANCOVA, *F*- and *p*-values are reported after controlling for FSIQ2 in parentheses

^f^ in seconds

^g^ National percentile rank

^h^ *F*(1,31)

Table S2. Correlation between brain measures in control and at-risk infants (collapsed across 6 and 12 months) and their language and nonlinguistic communication scores at 13 and 15 months

|  | | | |  | Language scores | | | Nonlinguistic communication | | | | | | | |
| --- | --- | --- | --- | --- | --- | --- | --- | --- | --- | --- | --- | --- | --- | --- | --- |
|  | | | |  | Words understood | Words produced | | Early gestures | | Late gestures | | | Total gestures | | |
| 13 months | | | | | | | | | | | | | | | |
| Controls (*n* = 25) | | | | | | | | | | | |  | | |  |
|  | | | Left MAs | | -.003^a^ (.987)^b^ | | .132 (.531) | -.254 (.221) | | -.108 (.608) | | | -.151 (.472) | | |
|  | | | Right MAs | | -.081 (.701) | | -.21 (.313) | -.036 (.864) | | -.006 (.979) | | | -.014 (.945) | | |
|  | | | Left durations | | .08 (.703) | | .24 (.249) | -.044 (.834) | | -.036 (.863) | | | -.03 (.888) | | |
|  | | | Right durations | | -.231 (.266) | | -.185 (.377) | -.099 (.637) | | -.279 (.178) | | | -.249 (.231) | | |
| At-risk (*n* = 25) | | | | | | | | | | | |  | | |  |
|  | | Left MAs | | | -.043 (.839) | | .091 (.664) | -.079 (.708) | | -.016 (.94) | | | -.056 (.791) | | |
|  | | Right MAs | | | -.156 (.456) | | -.102 (.628) | .047 (.825) | | .135 (.519) | | | .088 (.676) | | |
|  | | Left durations | | | -.204 (.328) | | .085 (.686) | .135 (.521) | | .032 (.88) | | | .037 (.862) | | |
|  | | Right durations | | | -.387 (.056) | | **-.506 (.01)*** | -.143 (.496) | | -.181 (.386) | | | -.225 (.279) | | |
| 15 months | | | | | | | | | | | | | | | |
| Controls (*n* = 25) | | | | | | | | | | |  | | |  | |
|  | Left MAs | | | | -.05 (.813) | | -.056 (.791) | | -.163 (.436) | | .008 (.971) | | | -.007 (.975) | |
|  | Right MAs | | | | -.123 (.56) | | -.258 (.213) | | -.06 (.775) | | -.008 (.971) | | | -.049 (.817) | |
|  | Left durations | | | | -.019 (.929) | | .056 (.789) | | -.014 (.947) | | .048 (.821) | | | .072 (.732) | |
|  | Right durations | | | | -.278 (.179) | | -.108 (.609) | | -.148 (.481) | | -.351 (.086) | | | -.355 (.081) | |
| At-risk (*n* = 25) | | | | | | | | | | |  | | |  | |
|  | Left MAs | | | | -.149 (.477) | | .131 (.532) | | -.093 (.659) | | -.392 (.052) | | | -.353 (.083) | |
|  | Right MAs | | | | -.027 (.898) | | -.093 (.658) | | -.069 (.744) | | .338 (.098) | | | .271 (.19) | |
|  | Left durations | | | | -.265 (.201) | | .039 (.854) | | -.111 (.598) | | -.173 (.408) | | | -.181 (.387) | |
|  | Right durations | | | | **-.423 (.035)*** | | -.375 (.065) | | -.087 (.68) | | -.177 (.397) | | | -.163 (.437) | |

^a^ Pearson correlation coefficients

^b^ *p*-values are given in parentheses

Parents were asked to complete the CDI (infant form: words and gestures; Fenson et al., 1993) on the day their child reached 13 and 15 months of age. Language comprehension was assessed with subscales words understood and words produced. Nonlinguistic communicative development was measured with early gestures (intentional communication), late gestures (representational skills) and total amount of gestures (early + late). Mean activations (MAs) and durations of auditory responses in the left and right hemisphere were collapsed across 6 and 12 months to increase statistical power and were then correlated with the percentiles of each CDI measure. Atypical durations in the right hemisphere in at-risk infants predicted words produced at 13 months and words understood at 15 months with longer durations linked to lower percentile of words understood at 13 months and words produced at 15 months (marked in **bold**). This was not the case for control infants.
